# Bromodomain and extra-terminal (BET) proteins target Moloney murine leukemia virus integration to transcription start sites

**DOI:** 10.1186/1742-4690-10-S1-O20

**Published:** 2013-09-19

**Authors:** Jan De Riick, Christine de Kogel, Jonas Demeulemeester, Sofie Vets, Nirav Malani, Frederic D Bushman, Katrien Busschots, Steven Husson, Rik Gijsbers, Zeger Debyser

**Affiliations:** 1 Laboratory for Molecular Virology and Gene Therapy, KU Leuven, Leuven, Belgium; 2 Department of Microbiology, University of Pennsylvania School of Medicine, Philadelphia, USA; 3 Biomedical Research Institute, University Hasselt, Diepenbeek, Belgium; 4 Systemic Physiological & Ecotoxicological Research, University of Antwerp, Belgium

## 

A hallmark of retroviral replication is stable integration of the viral genome in the host cell DNA. This characteristic makes retroviral-derived vector particles attractive vehicles for gene therapy. However, retroviral integration is not a random process. Lentiviruses preferentially integrate in the body of active transcription units, while gammaretroviruses, including Moloney Murine Leukemia Virus (MLV), favour transcription start sites and CpG islands. In clinical trials using gammaretroviral vectors for gene therapy, leukemogenesis has been associated with integration of vectors near oncogene transcription start sites. We found that the bromodomain and extra-terminal (BET) proteins (BRD2, BRD3 and BRD4) interact with MLV integrase and direct integration towards transcription start regions. BET proteins specifically bind and co-localize with the gammaretrovirus integrase protein in the nucleus of the cell. The interaction is gammaretroviral-specific and mediated by the integrase C-terminal domain and the BET extraterminal (ET) domain as determined by co-immunoprecipitation assays and in an Alphascreen assay using recombinant proteins. Interfering with chromatin interaction of BET proteins via specific bromodomain inhibitors JQ1 and l-BET decreases MLV virus replication and MLV vector transduction 5-to 10-fold, while HIV vector transduction is not affected. Analysis of viral DNA intermediates by quantitative PCR revealed a block at the integration step. In addition, bromodomain inhibitors do not have an effect on the late steps of viral replication. MLV integration site distribution analysis revealed a strong correlation with the BET protein chromatin binding profile. Finally, expression of an artificial fusion protein that merges the BET integrase binding domain with the chromatin interaction domain of the lentiviral targeting factor LEDGF/p75, retargets MLV integration into the body of actively transcribed genes, paralleling the Human Immunodeficiency Virus (HIV) integration pattern. Our results explain the molecular mechanism behind gammaretroviral integration site targeting and suggest methods for engineering gammaretroviral vectors with a safer integration site profile.

